# A Molecular Insight into Algal-Oomycete Warfare: cDNA Analysis of *Ectocarpus siliculosus* Infected with the Basal Oomycete *Eurychasma dicksonii*

**DOI:** 10.1371/journal.pone.0024500

**Published:** 2011-09-15

**Authors:** Laura Grenville-Briggs, Claire M. M. Gachon, Martina Strittmatter, Lieven Sterck, Frithjof C. Küpper, Pieter van West

**Affiliations:** 1 Aberdeen Oomycete Laboratory, University of Aberdeen, Aberdeen, United Kingdom; 2 Scottish Association for Marine Science, Scottish Marine Institute, Oban, Argyll, United Kingdom; 3 Department of Plant Systems Biology, Flanders Institute for Biotechnology (VIB), Ghent, Belgium; 4 Department of Plant Biotechnology and Genetics, Ghent University, Ghent, Belgium; University of Sydney, Australia

## Abstract

Brown algae are the predominant primary producers in coastal habitats, and like land plants are subject to disease and parasitism. *Eurychasma dicksonii* is an abundant, and probably cosmopolitan, obligate biotrophic oomycete pathogen of marine brown algae. Oomycetes (or water moulds) are pathogenic or saprophytic non-photosynthetic Stramenopiles, mostly known for causing devastating agricultural and aquacultural diseases. Whilst molecular knowledge is restricted to crop pathogens, pathogenic oomycetes actually infect hosts from most eukaryotic lineages. Molecular evidence indicates that *Eu. dicksonii* belongs to the most early-branching oomycete clade known so far. Therefore *Eu. dicksonii* is of considerable interest due to its presumed environmental impact and phylogenetic position. Here we report the first large scale functional molecular data acquired on the most basal oomycete to date. 9873 unigenes, totalling over 3.5Mb of sequence data, were produced from Sanger-sequenced and pyrosequenced EST libraries of infected *Ectocarpus siliculosus.* 6787 unigenes (70%) were of algal origin, and 3086 (30%) oomycete origin. 57% of *Eu. dicksonii* sequences had no similarity to published sequence data, indicating that this dataset is largely unique. We were unable to positively identify sequences belonging to the RXLR and CRN groups of oomycete effectors identified in higher oomycetes, however we uncovered other unique pathogenicity factors. These included putative algal cell wall degrading enzymes, cell surface proteins, and cyclophilin-like proteins. A first look at the host response to infection has also revealed movement of the host nucleus to the site of infection as well as expression of genes responsible for strengthening the cell wall, and secretion of proteins such as protease inhibitors. We also found evidence of transcriptional reprogramming of *E. siliculosus* transposable elements and of a viral gene inserted in the host genome.

## Introduction

Like all other living organisms, algae suffer from diseases, which may range from spectacular outbreaks in natural populations to significant losses in multibillion dollar crops such as nori [1]. As aquaculture continues to rise worldwide, and with algae considered as a sustainable biofuel source, pressure is mounting to design efficient disease control methods. More generally, parasites and pathogens are increasingly being considered of equal importance with predators for ecosystem functioning [2]. In aquatic as well as terrestrial environments, altered disease patterns in disturbed environments are blamed for sudden extinctions, regime shifts, and spreading of alien species. Likewise, algal pathogens exert a range of complex, profound, sometimes subtle, and often unexpected impacts in aquatic ecosytems. These range from host generation shifts to changes in biogeochemical cycling and atmosphere chemistry (e.g. [3]).

Despite mounting recognition of their importance, molecular knowledge on algal pathogens is hitherto restricted to viruses (reviewed in [1]). Likewise, the molecular characterisation of algal immune responses is in its infancy [4]. To help address these fundamental and ecological questions, we have developed a laboratory-controlled pathosystem involving the genome model seaweed *Ectocarpus siliculosus* [5] and the oomycete pathogen *Eurychasma dicksonii* [6], [7].

*Eu. dicksonii* is the most common and widespread eukaryotic pathogen of marine algae [8]. It occurs in all cold and temperate seas worldwide [9]. In culture, it infects virtually all species of brown algae tested (more than 40, [6]), including representatives of all major orders of this phylum. As brown algae are the predominant primary producers of temperate and polar rocky shores, and *Eu dicksonii* occurs in frequent epidemics [10], we infer that this pathogen contributes to shaping natural algal populations, therefore profoundly impacting ecosystem functioning [11].

Importantly, molecular taxonomy unambiguously designates *Eu. dicksonii* as the most early-branching clade within the oomycete lineage [7], [12]. Oomycetes (or water moulds) are classified within the group of Stramenopiles together with diatoms, golden-brown and brown algae [13]. They are secondarily non-photosynthetic organisms which exhibit either pathogenic or saprophytic lifestyles [14], [15]. In fact, many of the most devastating agricultural and aquacultural pathogens belong to the oomycetes [16], [17], [18], [19]. For example, *Phytophthora infestans*, the causal agent of potato late blight, is responsible for annual crop losses and pesticide costs exceeding £5 billion worldwide [20]. Many other species cause significant environmental damage, especially when recently introduced (e.g. *Phytophthora ramorum*). Therefore, the best-studied oomycete species are phylogenetically highly-derived plant pathogens, of which five fully sequenced genomes are hitherto available. However, pathogenic oomycetes are taxonomically much more diverse, and infect a remarkable palette of hosts ranging from marine algae, crustaceans, plants, nematodes, fungi, insects, to fishes and mammals [18]. From a physiological standpoint, *Eu. dicksonii* is typical of most basal oomycetes, inasmuch as it is an obligate biotroph, exclusively growing and propagating within a living host. After encysting at the surface of an algal cell, infectious spores inject their protoplasm into the host cytoplasm, and develop into a multinucleate coenocytic intracellular thallus. After having progressively filled the infected algal cell, the latter differentiates into a sporangium that releases new infectious zoospores [7]. The extremely broad host range of *Eu. dicksonii* is unusual among biotrophic pathogens, and may reflect a low level of specialization, suggesting that it may have retained ancestral infection mechanisms.

In summary, *Eu. dicksonii* is of considerable experimental interest because of its basal phylogenetic position, its presumed environmental impact, and amenability to molecular studies. Here, we set out to investigate the physiology of the *E. siliculosus* – *Eu dicksonii* host-pathogen interaction by constructing cDNA libraries of axenic cultures. We aimed to address what pathogenicity determinants and strategies *Eu. dicksonii* uses to infect *E*. siliculosus and what are the consequences of infection on algal physiology. Overall, we describe here a very original dataset that underlines both the taxonomical distance between *Eu dicksonii* and better-studied oomycetes, and the specialised marine lifestyle of this pathogen.

## Materials and Methods

### Biological material and microscopy

Axenic cultures of the fully sequenced *Ectocarpus siliculosus* strain CCAP 1310/4 and of *Eurychasma dicksonii* CCAP 4018/1 were obtained as described by [21]. They were maintained in half strength Provasoli medium [22] at 15°C, under illumination with daylight type fluorescent lamps at a 2–10 mE.m^−2^.s^−1^ irradiance and a 12 h photoperiod, as described by [6]. Being an obligate biotroph, *Eu. dicksonii* was maintained in co-culture with its *E. siliculosus* host and transferred into fresh medium every second week, together with some new uninfected algal host. The *Eu. dicksonii* CCAP 4018/1 strain was originally established from a single developing parasitic thallus, which was propagated into a clonal healthy algal host. For microscopy, infected algae were fixed for 45 min with 4% paraformaldehyde in microtubule-stabilising buffer [23], dipped into methanol for 30 s, and transferred into a DAPI solution (10 mg/mL in sterile seawater) for 5 minutes and observed under an epifluoresence microscope equipped with the following filter set: L365 FT 395, LP 420.

### Construction of cDNA libraries and sequencing strategy

Fifty *E. siliculosus* cells each containing a developing intracellular *Eu. dicksonii* thallus were dissected under the stereomicroscope using a glass Pasteur pipette, and transferred into RNALater®. Total RNAs were extracted, subjected to reverse-transcription using a poly-dT oligonucleotide, PCR-amplified, and cloned directionally into a pBluescript II sk+ vector (Vertis Biotechnologie AG, Germany). After plating, 3000 colonies were robot-picked. Their insert was further sequenced from both ends using Sanger technology (Genoscope, France).

In parallel, two densely infected *E. siliculosus* cultures (∼20 mg FW each) were harvested in RNALater®, without prior dissection. The “young” culture (4 weeks after inoculation) contained all *Eu. dicksonii* development stages, including young intracellular thalli, mature dehiscent sporangia and encysted spores at the surface of algal cells. In contrast, the “old” culture (2 months after inoculation) predominantly contained mature dehiscent sporangia and encysted spores. Total RNA was extracted and subjected to cDNA synthesis as above. For each sample, 50,000 sequence reads were obtained using 454 pyrosequencing.

### Bioinformatic analysis

#### Data assembly

The sequence reads were first assembled separately for each individual dataset using CAP3 (default parameters) [24], and then further assembled, also using CAP3, (default parameters, without resorting to quality file) into the final hybrid assembly discussed throughout the manuscript. Unigenes containing poor quality sequence reads (manual assessment of quality) were removed from the final dataset. A total of 5889 unassembled EST sequences (dissected library) were submitted to the EST db at EMBL and were assigned accession numbers FR839767-FR845655. Pyrosequencing reads (old culture and young culture libraries) were submitted to the EMBL short-read assembly (SRA) database. Sequences from each of the three databases were assembled into a total of 9847 unigenes.

#### Sequence Analysis

Unigenes were mapped to the *E. siliculosus* nuclear, chloroplastic and mitochondrial genomes with the use of GenomeThreader [25] to produce the ‘host’ dataset. GenomeThreader was applied with default parameters, except for the minimum alignment length and percent identity, which were set to 90% and 95% resp. Non-mapping unigenes were assigned to *Eu. dicksonii* after blastn analysis (98% to 100% sequence similarity) to remove potential contaminants, producing the ‘pathogen’ dataset. Open reading frames were predicted with OrfPredictor [26], based on a blastx analysis run against the NCBI Genbank non- redundant (nr) database and the *E. siliculosus* genome database (http://bioinformatics.psb.ugent.be/genomes/view/Ectocarpus-siliculosus). Sequence similarity searches were carried out using local blast tools [27], the NCBI nr database (28-07-2010 version) and several predicted proteomes of fully sequenced Eukaryotes. These include the proteomes of six oomycete species sequenced to date: *Phytophthora infestans,* [28]; *Phytophthora sojae,* [14]; *Phytophthora ramorum,* [14]; *Pythium ultimum,* [29]; *Hyaloperonospora arabidopsidis* [30] and *Saprolegnia parasitica* (draft genome, publically available at; (www.broadinstitute.org/annotation/genome/Saprolegnia_parasitica/MultiHome.html). Additional proteomes included were: the model brown alga and host species used in the current study, *Ectocarpus siliculosus*; the marine centric diatom *Thalassiosira pseudonana;* the marine pennate diatom *Phaeodactylum tricornutum*; the coccolith-bearing haptophyte *Emiliania huxleyi;* the Apicomplexan malaria parasite *Plasmodium falciparum;* the model plant *Arabidopsis thaliana*; the amoeboflagellate *Naegleria gruberi*; the amphibian pathogenic chytridiomycete fungus *Batrachochytrium dendrobatidis* and the plant pathogenic ascomycete fungus *Mycosphaerella fijiensis*. Predicted protein domains were identified using a standalone InterProScan program [31] (default settings) and the NCBI conserved domain database [32]. *E. siliculosus* transposable elements were identifed and named according to [5] and [33]. Their sequences were retrieved from the following URL: http://urgi.versailles.inra.fr/Data/Transposable-elements/Ectocarpus.

The Emboss tool infoseq [34] was used to generate statistics for host and pathogen datasets independently.

Signal peptide predictions were carried out using SignalP version 3.0 [35] with a hidden Markov model probability cutoff of 0.6, and 1160 candidate secreted proteins were obtained.

#### Effector protein motif predictions

Searches for RXLR and CRN motifs were carried out according to the methods of [36]. Briefly, string searches were carried out using the expression RxLR –x (1,40)- [ED] [ED] [KR] using custom build python scripts. This string search was also performed just with the RxLR expression, and was performed on all sequences, as well as those with predicted signal peptides. String searches were also performed with both the CRN motifs from *Phytophthora* species and *Pythium ultimum.* HMM profile searches for all these motifs were also carried out using the HMMer 2.2 package as described in [36].

Multiple alignments were conducted using the program CLUSTALW [37] (default parameters) and visualised using Geneious (version 4.8; [38]). Distance trees were constructed using Geneious with neighbour joining algorithms using 1000 bootstrap replications.

## Results

### Cytological observations of *Eu. dicksonii* infected *E. siliculosus*

**Figure 1:**
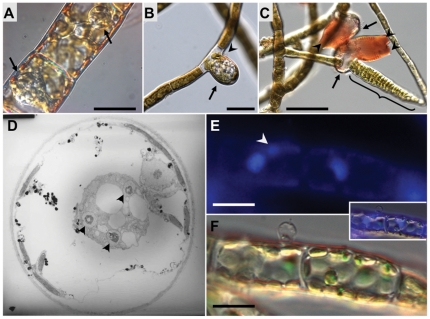
Co-localisation of *E. siliculosus* nuclei and *Eu. dicksonii thalli*. A, B, C: Successive stages of *Ectocarpus* infection by *Eurychasma dicksonii*. The *Eu. dicksonii* thallus develops as a spherical intracellular syncitium (A, arrows), progressively filling any individually infected algal cell, ultimately causing its hypertrophy (B, arrow). The syncitium then differentiates into a sporangium (C, arrows, Congo red staining) that releases new infectious spores into the medium via its apical apertures (C, arrowheads). The original infectious spore at the surface of the infected *Ectocarpus* cell is visible in B (arrowhead). The structure designated with a brace in C is an algal plurilocular sporangium containing parthenogenetic zoospores. Bars: A, B: 20 mm; C: 50 mm. D. *Eu. dicksonii* syncyitium (3 nuclei visible, arrowheads) developing next to the nucleus of a highly vacuolated *E. siliculosus* cell (strain CCAP 1310/299). Picture: courtesy of Dr S. Sekimoto. E & F. DAPI staining (E) and corresponding Nomarski image (F) of the microscopic sexual development stage (gametophyte) of the kelp *Macrocystis pyrifera* infected with *Eurychasma dicksonii*. The left hand side algal cell contains a very young *Eurychasma* thallus (one nucleus visible, white arrowhead) derived from the protoplasm of the empty spore visible at its surface. Insert: merged images. Scale bar: 10 um.

The morphological development and ultrastructural cytology of *Eu. dicksonii* infecting both *E. siliculosus* and the related filamentous phaeophyte alga *Pylaiella littoralis* has been investigated previously [7]. Infection is initated by secondary cysts, which attach to the surface of the host cell. Upon germination, the algal cell wall is breached, and cytoplasm from the pathogenic cell is transfered into the host cytoplasm. The initial phase of parasite development as a non-walled thallus is reminiscent of fungal pathogens of higher plants, such as *Plasmodiophora brassicae* and *Olpidium brassicae* (Figure 1A, [7]). The *Eu. dicksonii* thallus develops as a spherical intracellular syncitium, progressively filling the individual algal cell and ultimately causing its hypertrophy (Figure 1B). The sporangium develops by differentiation of the syncitium that releases new infectious zoospores (Figure 1C). The *Eu. dicksonii* thallus never propagates to other cells within the algal filament. During infection, the host nucleus and Golgi apparatus are closely associated with the pathogen thallus (Figure 1D–F).

### cDNA library quality control and statistics

**Figure 2:**
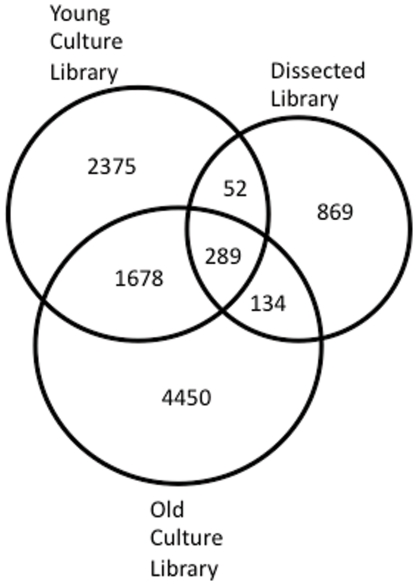
Venn Diagram showing numbers of overlapping sequences from each library within the total dataset.

3000 clones from microdissected *Eu. dicksonii-*infected *E. siliculosus* cells were Sanger-sequenced (Genoscope, France) and assembled into contigs (CAP3). This resulted in the identification of 1424 unigenes. Pyrosequencing reads, from both the ‘young culture’ and ‘old culture,’ were assembled at the NERC National Molecular Genetics Facility (Liverpool, UK), resulting in two data sets of 4778 unigenes (young culture) and 6840 unigenes (old culture). Unigenes from these three datasets were combined to produce a single dataset, and assembled to produce 9873 unigenes totalling over 3.5 Mb of sequence data (Table 1). 28 sequences (15 from the young culture library, 12 from the old culture library, and 1 singlet from the dissected library) were discarded on the basis of poor sequence (containing regions of more than 20 bases called as Ns). 38 singlets, originating from the dissected library, were also removed on the basis of containing less than 90 nt of readable sequence. The final total hybrid assembly (discussed throughout the text) of 9873 unigenes therefore contained 9658 contigs and 215 singlets. The total 9873 unigene dataset was made up of 295 contigs with representatives from all three libraries, 1680 contigs with representatives in both the young culture and old culture libraries, 52 contigs with representatives in both the young culture and dissected libraries, 136 contigs with representatives in both the dissected and old culture library and 7710 unigenes that were only present in one of the three libraries (Figure 2).

**Table 1:** Assembly statistics, individual cDNA libraries and final hybrid assembly.

	unigenes (average length; max length)	*Ectocarpus*: *Eurychasma* ratio	Non-redundant sequence information
**Dissected library**	**1432** (722 nts, 2085 nts)	30%: 70%	833 kb
**“Young” culture**	**4780** (273 nts, 3645 nts)	79%: 21%	1.4 Mb
**“Old” culture**	**6842** (298 nts, 2807 nts)	74%: 26%	2.1 Mb
**Hybrid assembly**	**9873** (350 nts, 5341 nts)	69%: 31%	> 3.5 Mb

Unigenes were mapped to the *E. siliculosus* genome as described in [5]. 6787 unigenes were mapped to the *E. siliculosus* genome and, therefore, predicted to be of host origin. 3152 unigenes did not map to the *E. siliculosus* genome. 66 poor quality or short sequences were excluded, leaving 3086 sequences ascribed to *Eu. dicksonii*. This resulted in the creation of two datasets, ‘host sequences’ containing 6787 unigenes (Figure S1), and ‘pathogen sequences’ (Figure S2) containing 3086 unigenes. Strikingly, unigenes from the host dataset have a mean GC of 51.9% whereas those from the pathogen dataset have a much lower mean GC of 40.5% (Table 2).

**Table 2:** Characteristics of host and pathogen datasets.

	Total Dataset	Host sequences	Pathogen sequences
**Total unigenes**	9873	6787	3086
**Longest contig (nt)**	5341	4513	5341
**Shortest contig (nt)**	90	95	92
**Mean contig length (nt)**	355	325	409
**Mean GC (%)**	48.2	51.9	40.5*
**Predicted secreted proteins**	1190 (11.9%)	839 (12.4%)	351 (11.6%)

### Highly expressed transcripts

#### Major up-regulation of Ectocarpus transposable elements

**Figure 3:**
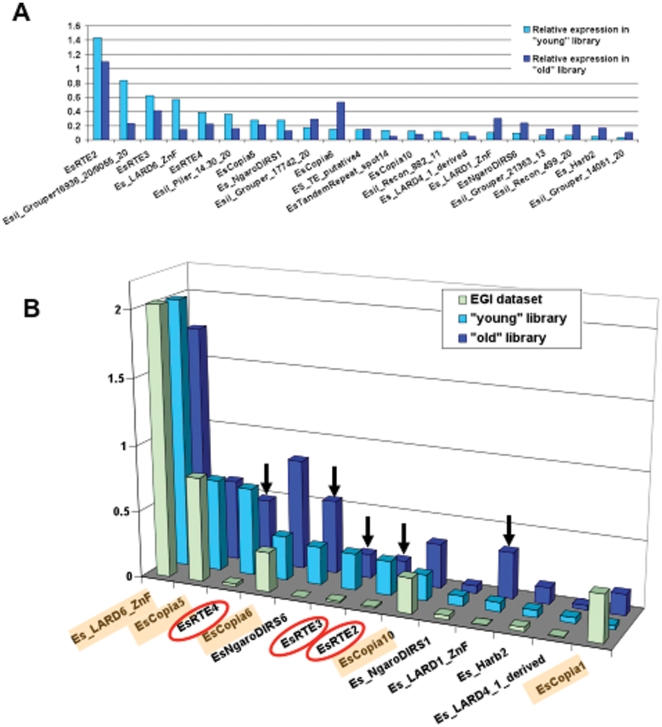
Over-representation of *Ectocarpus* transposable elements (TEs) in *Eurychasma*-infected libraries. **A.** Relative expression of *Ectocarpus* TEs in the pyrosequenced “young” and “old” *Eurychasma-*infected libraries. Total read counts were normalised over contig length in order to identify the most expressed unigenes in each library. Expression values are given for all TEs belonging to the top 150 most highly expressed unigenes in at least one library. If several contigs matched the same TE in a given library, their relative expression levels were summed. TE nomenclature is as per Maumus (2009). **B.** Differential expression pattern of *Ectocarpus* TEs between the pyrosequenced “young” and “old” *Eurychasma-*infected libraries vs. the Ectocarpus Genome Initiative EST collection (“EGI dataset”, Cock *et al*, 2010). For each library, sequence read counts were normalised over the genome coverage of each TE in order to control for the leaky transcription hypothesis. The 5 most highly expressed TEs in the EGI dataset are highlighted in orange; those circled in red belong to the 10 most abundant repeated elements identified in the *Ectocarpus* genome. Arrows point to TEs highly induced in the “young” and “old” infected cultures.

We used the number of sequence reads normalised over contig length as a proxy to evaluate relative gene expression levels in both the “young” and “old” pyrosequenced libraries. Unsurprisingly, the vast majority of the most abundantly expressed transcripts are host housekeeping genes. However, 16 (resp. 25) of the top 150 most expressed unigenes in the young and old libraries are *E. siliculosus* transposable elements (TEs*). E. siliculosus* TEs identified among the top most 150 highly expressed contigs in at least one library were extracted, and their expression level is plotted on Figure 3A. We further compared the expression level of TEs annotated by [5] between the two *Eu. dicksoniii*-infected libraries and the *Ectocarpus* genome initiative EST collection (Figure 3B). The latter mostly encompasses distinct development stages of unstressed *Ectocarpus*. Whereas pyrosequencing read counts and EST numbers are not directly comparable between the EGI dataset [5] and ours, differential expression of some TEs is nevertheless clearly discernible. Indeed, the five most expressed TEs identified by Cock and co-authors are also well represented in both *Eu. dicksonii-*infected libraries. However, the retroelements RTE2, RTE3 and RTE4, the LTR element NgaroDIRS6, and the LARD element EsLARD1_ZnF, virtually silent in unstressed conditions, appear strongly induced in the “young” and “old” pyrosequenced libraries. Interestingly, RTE2, RTE3 and RTE4 also belong to the top 10 most abundant repeats in *the E. siliculosus* genome, suggesting stress-induced transposing activity in the *E. siliculosus* genome [33].

#### Highly expressed Eurychasma transcripts

The 10 most abundant sequences within the dissected library are of pathogen origin, with the exception of a conserved unknown *E. siliculosus* protein, demonstrating the efficacy of microdissection for enriching the sample in pathogen RNAs (Table 3). The majority of the most abundant sequences are housekeeping and ribosomal genes. However, three unigenes of *Eu. dicksonii* origin are highly abundant in this library and are proteins with unknown functions (Table 3). Contig500203 encodes a methionine and lysine rich unknown protein. Contig500580 encodes a predicted protein with similarity to a group of unknown oomycete proteins. These proteins are a group of at least 8 proteins present in the *Saprolegnia parasitica* genome, but absent in the genomes of other sequenced oomycetes. These appear to be uncharacterised cytoplasmic proteins, which may be ancestral proteins lost from higher oomycetes. Contig50031 appears to encode a lysine and alanine rich protein or protein fragment, with no similarity to known proteins.

**Table 3:** Top 10 most highly expressed unigenes in the Eu. dicksonii-enriched microdissected library.

Contig	Length (bp)	Members	Origin	Hybrid assembly	Top blastn/x hit custom database* or Genbank nr
500130	93	80	Pathogen	In contig2041	PITG_00179 40S ribosomal protein S12 *P. infestans* 6e-44
500773	1473	65	Pathogen	In contig2006	XP_002908783.1 beta 1 tubulin *P. infestans* e = 0
500203	831	49	Pathogen	In contig1846	No hit
5001047	1701	47	Pathogen	5001047	18S rRNA *E. dicksonii*
500580	1176	43	Pathogen	In contig1954	SPRG_15343T0 predicted protein *S. parasitica* 4e-10
500569	756	40	Pathogen	In contig1953	XP_001809251 similar to I-connectin *T. castaneum* 7e-12
500393	1442	33	Pathogen	500393	28S rRNA *E. dicksonii*
500904	937	28	Pathogen	In contig2041	PITG_00179 40S ribosomal protein S12 *P. infestans* 6e-44
500588	1258	26	Host	500588	Esi0110_0040 conserved hypothetical protein
50031	713	24	Pathogen	In contig1807	No hit

*custom database made up of proteomes described in Figure 5.

### Functional Annotation

The 9873 unigene set was annotated by comparison to the NCBI non-redundant (nr) protein database (28-07-2010 version) and the *E. siliculosus* genome database (http://bioinformatics.psb.ugent.be/webtools/bogas/overview/Ectsi; [5] using BLAST analyses [25]. Overall, approximately 20% of the sequences in the total dataset showed similarity to previously described genes in the NCBI nr protein database, using an e value cutoff of <1e-05, indicating that this is largely a unique dataset.

#### Functional annotation of host genes

**Figure 4:**
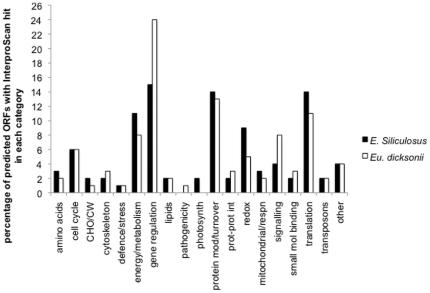
Classification of unigenes based on identification of functional domains using InterproScan. Functional categories assigned to the 822 *E. siliculosus* (host) and 1397 *Eu. dicksonii* (pathogen) predicted ORFs (12% and 43% of total unigenes, respectively) with an Interpro hit. Numbers assigned to each category are percentages of the 822 and 1397 predicted ORFs. Amino acids includes amino acid synthesis and metabolism; cell cycle genes also include mitosis, DNA and RNA synthetic genes; CW/CHO includes cell wall biosynthesis and carbohydrate synthesis and metabolism; cytoskeleton includes transcripts involved in cytoskeletal rearrangements; defence and stress includes transcripts involved in general and specific defence or stress responses; energy/metabolism includes transcripts involved in energy production and cellular metabolism but does not include those involved specifically in respiration; gene regulation includes transcripts involved in DNA and RNA binding, transcription factors and transcripts with coiled-coil domains, lipids includes both lipid biosynthesis and metabolism; pathogenicity includes transcripts with a predicted function in pathogenicity or virulence, photosynth includes photosynthetic machinery; protein mod/turnover includes transcripts involved in protein folding, targeting, modification and degradation; prot-prot int includes transcripts predicted to be involved in protein-protein interactions; redox includes transcripts involved in homeostasis, detoxification and those classified as redox antioxidants; mitochondrial/respn transcripts are predicted to be mitochondrial and/or involved in respiration; signalling includes transcripts with a predicted role in signalling or signal transduction; small mol binding includes those transcripts which bind small molecules; translation includes those transcripts with a role in translation, including ribosomal proteins; transposons includes sequences both of transposons and those of putative retroviral origin; transcripts which could not be assigned to any one of the above categories are classified in the section termed other.

The *E. siliculosus* unigene set (host sequences) was initially annotated by direct comparison to the *E siliculosus* genome sequence [5]. Blastx analysis (expectation value <1e -05) of this dataset to the *E. siliculosus* predicted proteome resulted in 1299 (19.2%) of sequences matching predicted *E siliculosus* proteins. 46% (3163) have a significant blastn hit to genomic mRNA sequences, which include the 3′ UTRs, with the remainder of the sequences hitting mitochondrial, chloroplastic or intergenic sequences. Protein sequences were predicted using ORFPredictor [26] using the *E. siliculosus* blastx results and checked for agreement with the *E. siliculosus* genome database. Predicted protein sequences were analysed for the presence of an N-terminal signal sequence, using both Hidden Markov Models and Neural Network algorithms in SignalP [35]. 839 (12.4%) of the host unigenes were predicted to contain a signal peptide. Predicted proteins were also analysed for functional domains using InterProScan [31]. 822 unigenes (12%) of the dataset had one or more hits to functional domains using InterProScan. All the 822 predicted ORFs with InterproScan hits were within the 1299 sequences predicted to match protein-coding regions of the *E. siliculosus* genome. 63% of the unigenes that are derived from *E. siliculosus* protein coding regions, therefore, contain functional domains as identified by InterproScan. Functional annotation of host genes was checked with the HECTAR predictions for full-length sequences in the *E. siliculosus* genome database [5]. Predicted ORFs in this dataset were assigned to functional categories based on GO annotations of the InterProScan/HECTAR predictions (Figure 4). The largest category was gene regulatory proteins (15% of the total), including predicted transcription factors, translation factors, DNA and RNA binding proteins and proteins containing one or more coiled coil domains. 14% of predicted ORFs were classified as involved in translation, with approximately two thirds of these being ribosomal proteins. 14% of predicted proteins were also classified as being involved in protein modification, targeting or turnover including chaperones, and proteins involved in ubiquitination and proteolysis.

#### Functional annotation of pathogen genes

The *Eu. dicksonii* unigene dataset (containing sequences that did not map to the host genome) was analysed and ORFs were predicted as described above for *E. siliculosus*.

InterProScan analysis was performed *on Eu. dicksonii* predicted ORFs, in the same manner as on the host dataset. 1397 of the 3086 (44%) pathogen unigenes were predicted to contain one or more functional domain using InterProScan. As seen with the host dataset, the largest class of proteins were those predicted to contain gene regulatory domains, including those with coiled-coil domains. This class represented almost a quarter (24%) of all pathogen sequences with functional domains (Figure 4). 8% of sequences were classified as being involved in signalling in the pathogen dataset, compared to just 4% of host sequences. Interestingly, proteins predicted to be involved in pathogenicity and suppression of immunity were also identified in the pathogen dataset, but were absent from host sequences. A higher percentage of proteins classified as having a role in redox reactions, including those involved in detoxification and homeostasis, were identified within the host dataset (9%) in comparison to the pathogen dataset (5%). It is anticipated that several of these proteins function in cellular defence mechanisms.

**Figure 5:**
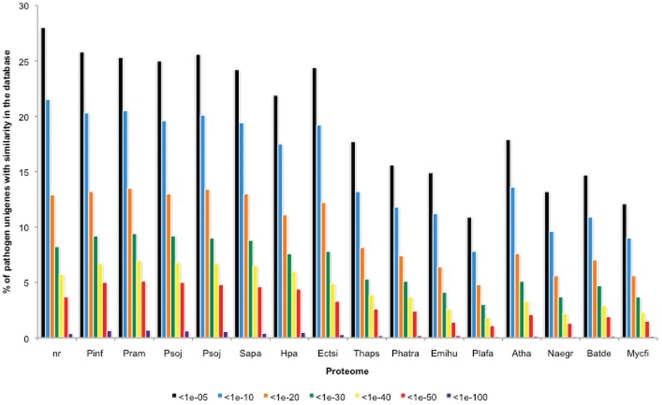
Comparison of *Eu. dicksonii* sequences to the non-redundant protein database at NCBI and proteomes of fully sequenced organisms. Unigenes were blasted using the BLASTX algorithm to the nr database and to the proteomes of *Phytophthora infestans* (Pinf), *Phytophthora ramorum* (Pram), *Phytophthora sojae* (Psoj), *Pythium ultimum* (Pult), *Saprolegnia parasitica* (Sapa), *Hyaloperonospora arabidopsidis* (Hpa), *Ectocarpus siliculosus* (Ectsi), *Thalassisosira pseudonana* (Thaps), *Phaeodactylum triconutum* (Phatr), *Emiliana huxleyi* (Emilhu), *Plasmodium falciparum* (Plafa), *Arabidopsis thaliana* (Atha), *Naegleria gruberi* (Naegr), *Batrachochytrium dendrobatidis* (Batde) and *Mycosphaerella fijiensis* (Mycfi). For each E-value class the percentage of unigenes showing similarity is indicated.

In addition to blastx similarity searches with the NCBI nr database, fifteen eukaryotic proteomes (listed in the methods section) were added to the analysis (Figure 5). Up to 25% of the pathogen sequences showed significant similarity (< 1e-05) to proteins from sequenced oomycetes. Interestingly about the same number (28% at <1e -05) showed significant similarity to proteins in the NCBI nr dataset and to the algal host *E. siliculosus* predicted proteome (24.4% at 1e-05), indicating that this dataset is largely made up of unique, uncharacterised sequences. As expected, the *Eu. dicksonii* dataset showed the most similarity to the oomycete proteomes, and to *E. siliculosus*. A lesser degree of similarity was seen with the diatom, haptophyta and apicomplexa proteomes. Surprisingly, a higher level of similarity (up to 18%, at 1 e-05) was observed between the pathogen unigenes and the proteome of the model plant *Arabidopsis thaliana*, than with some of the chromalveolata, such as *Plasmodium falciparum* with which there was only 11% similarity (at 1e-05). Likewise, up to 15% sequence similarity was seen between *Eu. dicksonii* unigenes and distantly related organisms such as the amoeboflagellate *Naegleria gruberi* and true fungi (Figure 5).

351 (11.6%) of the pathogen sequences were predicted to contain a signal peptide. 179 of these also had one or more hits to InterProScan domains, including non-specific ‘seg’ hits. At least 30 of those containing known domains were in fact membrane proteins, or fragments of membrane proteins that were likely picked up with the SignalP algorithms because they are not full length. In the majority of cases, it is not possible to conclude whether our contigs represent full-length sequences with real signal peptides, or simply fragments of proteins with hydrophobic or transmembrane spanning regions. However, 10 sequences from the *Eu. dicksonii* dataset are predicted to encode full-length, unknown, secreted proteins that do not contain transmembrane domains (Table 4). These predicted proteins either have no sequence similarity to known proteins or domains, or have an Interpro hit to the non-specific ‘seg’ database. There is no significant sequence similarity between the 10 predicted proteins. However, three of them have similarity to uncharacterised secreted proteins from other oomycetes. These proteins may possibly be ancient oomycete effectors, however further work is required to investigate the precise function of these proteins. Other potentially secreted pathogenicity factors, which contain conserved domains are listed in Table S1.

**Table 4:** Predicted full-length secreted Eu. dicksonii proteins of unknown function.

Contig	Length (aa)	Signal Peptide (aa)	Top blastx hit custom database* or Genbank nr
1471	154	22	no hits
1573	91	41	conserved hypothetical (134aa) protein *Anaerococcus tetradius* ZP_03930892 2e-12.
908	132	34	no hits
1962	140	18	conserved hypothetical (102aa) protein *Phytophthora infestans* PITG_08303 1e-10.
1984	231	15	no hits
402981	113	21	conserved hypothetical (115aa) protein *Phytophthora infestans* PITG_08745 0.01e.
500818	138	15	no hits
500933	130	29	no hits
405461	157	24	hypothetical protein Psta_475a (266aa) *Pirellula staleyi* YP_003373260 5e-08.
4YO14FM1	141	34	no hits

*custom database made up of proteomes described in Figure 5.

### Insights into oomycete infection of brown algae

#### Pathogen cell surface proteins

Mucins are typically high molecular weight cysteine, threonine, or serine non-glycosylated proteins that act as lubricants, protectants, signal tranducers or adhesins [39] and have been identified in the cell walls of higher oomycetes such as *P. ramorum* [40] and *P. infestans* [41]. We identified three gene fragments (contig1838, contig403969 and contig500361) with similarity to mucins or mucin-like sequences in the pathogen dataset (Table S1). We also identified a unigene sequence, contig500361, from the dissected library with a fibronectin type-3 domain (Table S1). Contig500361 is missing an initial start codon, but the entire protein fragment is predicted to be on the outside of the cell, anchored to the cell membrane, using both the new Eukaryotic subcellular predictor, Euk-mPLoc 2.0, [42] and TMHMM [43]. Contig500361 is not predicted to contain a signal sequence for secretion, according to SignalP [33] however it is likely that the N-terminal section of the protein is missing from Contig500361. Fibronectin is a high molecular weight extracellular matrix protein with diverse functions including roles in adhesion, growth, differentiation and wound healing [44]. Contig500361 is, therefore, likely to be part of a large surface protein in *Eu. dicksonii.*

#### Host and pathogen cell wall interactions

Higher oomycetes secrete cell wall degrading enzymes, to aid in penetration of host cells [45]. Since *Eu. dicksonii* penetrates the host algal cell to initiate infection, we looked for cell wall degrading enzymes within the *Eu. dicksonii* unigenes, as well as for algal genes that may be involved in strengthening or remodelling the host cell wall to prevent penetration.

**Figure 6:**
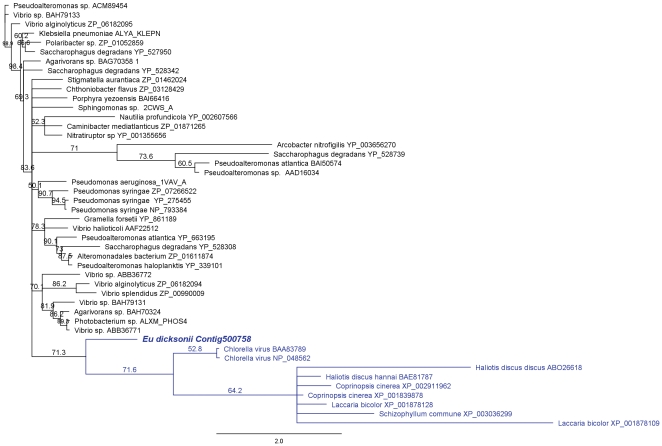
A *Eu. dicksonii* candidate pathogenicity effector has similarity with alginate lyases of brown algal grazers and fungi. The conserved amino acid sequence region between eukaryotic, bacterial and viral alginate lyases present in the NCBI non-redundant database and the predicted protein sequence of the *Eu. dicksonii* gene fragment Contig500758 were aligned using MUSCLE. A dendrogram was produced using Geneious 4.8 with a neighbour-joining algorithm and 1000 bootstrap iterations. Branches with less than 50% bootstrap support were collapsed. Genbank accession numbers of the sequences used in the alignment are indicated on the tree. The *Eu. dicksonii* sequence groups with a clade of eukaryotic alginate lyases from fungi and abalone (*Haliotis* sp.), highlighted in blue. The latter also contains sequences from a green algal virus, thought to have been acquired by horizontal gene transfer.

An *Eu. dicksonii* unigene had similarity to genes involved in the degradation of alginates, which are a key component of the brown algal cell wall matrix [46]. Contig500758 was predicted by InterproScan to contain a full alginate lyase 2 domain and has weak similarity to a 222 aa alginate lyase from *Pseudomonas syringae* pv. *phaseolicola* (Table S1). Phylogenetic analysis showed that it also had similarity to alginate lyase sequences from *Chlorella* virus and algal gastropods (Figure 6). However, it is likely that this gene is not full length, and therefore the prediction of signal peptides, specific substrates or catalytic residues is speculative without first obtaining the full length gene sequence. Interestingly, the *Hyaloperonospora arabidopsidis, Saprolegnia parasitica, Phytophthora infestans, Phytophthora ramorum* and *Phytophthora sojae* genomes do not contain homologues of Contig500758, indicating that this may be uniquely produced for the degradation of the algal host wall by *Eu. dicksonii*, and thus directly relate to its host spectrum. Contig500758 has similarity to alginate lyases from algal grazers (*Haliotis* species) and fungi and groups within a clade of alginate lyases from these organisms (Figure 6).

We also identified a mannuronan C5-epimerase, the enzyme that catalyses the final step in alginate biosynthesis in the *E. siliculosus* sequences (Contig290, *E. siliculosus* Esi0495_0002). In kelps, C5 epimerases belong to a small family, with some isoforms induced in protoplast and elicitor-treated cultures [47]–[48]. The representation of the otherwise lowly expressed Esi0495_0002 gene [5] in our dataset suggests that alginate biosynthesis may be important to strengthen the host cell wall as a response to *Eu. dicksonii* infection. However, none of the other *Ectocarpus* enzymes involved in alginate biosynthesis [49] were represented in our dataset.

Although not full length, two additional *Eu. dicksonii* contigs encode glucanases that may be involved in breakdown of host cell wall glucans or cellulose. The predicted protein sequence of Contig500983 contains a partial exo-beta-1,3-glucanase domain (Table S1). It has similarity to glucan 1,3 beta-glycosidase and endo-1,3-beta glucanase from *P. infestans*, both of which are secreted proteins. Contig403087 is similar to the secreted putative exo-1,3-beta-glucanase from *P. infestans* (Table S1).

There are 9 putative cellulose synthase genes in *E. siliculosus* [5]. We identified three of these in our host dataset, suggesting a role for host cellulose synthesis during infection by *Eu. dicksonii*. Contig1852 mapped to host gene Esi0004_0105, Contig404144 mapped to host gene Esi0185_0053, whilst Contig301614 and Contig1623 mapped to the 3′ untranslated region of Esi0231_0017 (Table S1).

*Eu. dicksonii* thalli are first formed in a non-walled state, and then mature to walled thalli as infection progresses [7]. Therefore we expect cell wall biosynthesis to be an important part of the infection process of this oomycete. Oomycetes have traditionally been described as cellulosic organisms, however, chitin and chitosaccharides have been identified in the cell walls of several oomycetes, as have chitin synthases [50], [51]. We were not able to identify genes with similarity to cellulose synthases in *Eu. dicksonii.* However, we did identify a putative chitin synthase, namely Contig500448, which displays significant similarity to the chitin synthase 1 gene from the oomycete *Saprolegnia monoica* [52], [53] (Table S1).

Brown algal cell walls also contain sulfated fucans (fucoidans). Possible enzymes in the biochemical pathway for the synthesis and sulfation of fucans have recently been identified in the *E. siliculosus* genome [49], however, we were unable to identify any of these enzymes in our *E. siliculosus* dataset. At least two kinds of glycosidases act on fucans, flucan sulfate hydrolases and fucoidonase [54] however we were unable to detect pathogen transcripts with similarity to these enzymes in our dataset.

#### Searching for known oomycete effector protein families

All oomycete avirulence genes cloned to date contain a signal peptide for secretion and the RXLR amino acid motif at the N terminus of the protein. Hundreds of effector molecules containing this motif have been predicted in the genomes of sequenced oomycetes [28]. We therefore mined our dataset for RXLR-like sequences. We were unable to unambiguously identify transcripts that fulfill the criteria for putative RXLR effectors, as identified by [55] and [28].

The Crinkler (CRN) protein family elicits host responses in *P. infestans* host interactions, and contain an LFLAK motif. These effector proteins have been identified in all *Phytophthora* species sequenced to date [14], [28] and in the legume pathogen *Aphanomyces euteiches* [36]. A group of similar proteins has also been identified in the *Pythium ultimum* genome, with a related, but divergent, motif [29]. It is therefore possible that CRN proteins are ancestral effector proteins present throughout the oomycete lineage. However, we were unable to identify CRN proteins in our dataset. Thus, complete transcriptome or full genome sequencing will be needed to prove or disprove the absence of RXLR and CRN effectors in *Eu. dicksonii*.

#### Genes encoding other potential pathogenicity factors in Eu. Dicksonii

A vast array of pathogenicity factors and potential effector molecules, which may be involved during infection, or which trigger host defences, have now been identified in higher oomycete pathogens [16], [56]. Pathogenicity determinants are, either, presented at the cell surface, or secreted and/or actively transported into the host cell, to manipulate the host. We therefore, mined our dataset for potentially secreted proteins with similarity to known effectors or pathogenicity factors.

Contig400638 encodes a protein with a predicted protein tyrosine phosphatase-like (PTPLA) domain and a signal peptide for secretion. It has significant similarity to a conserved hypothetic protein from *P. infestans* and to a protein tyrosine phosphatase-like protein from *Rattus norvegicus* (Table S1). PTPLA proteins are key regulatory proteins, involved in regulating signal transduction by removal of phosphate from tyrosine residues in proteins such as MAP kinases. It is, therefore, possible that *Eu. dicksonii* secretes a PTPLA protein to interfere with host defence signal transduction, perhaps blocking transduction of host signaling that would lead to an algal defence response.

Contig500988 encodes a protein with a signal peptide and a histone deacetylase domain. The predicted protein has significant similarity to histone deacetylases from protozoan parasites (Table S1). Histone deacetylases may be involved in transcriptional regulation by removal of acetyl groups from the lysine residues of histones.

Sequences derived from *Eu. dicksonii* with similarity to cyclophilins/immunophilins, which may also be involved in pathogenicity were also identified (Table S1).

#### Eu. dicksonii genes involved in growth regulation and programmed cell death

Several *Eu. dicksonii* sequences, which are not predicted to be secreted, or which are not full-length, are similar to genes involved in growth inhibition or programmed cell death in other models. Singlet 8YN09FM1 encodes a protein fragment which contains a PHD domain (Table S1). PHD folds into an interleaved type of Zn-finger chelating Zn ions in a similar manner to RING-finger domains. Several PHD-finger proteins have now been identified that bind modules of methylated histone H3 and thereby inhibit transcription [57], [58], [59]. 8YN09FM1 also contains an inhibitor of growth domain (Table S1), which binds chromatin and acts as a transcription regulator. 8YN09FM1 does not encode a full-length protein, and does not contain the predicted start of the protein. It is therefore not possible to determine if this protein is secreted to interfere with host growth during infection, or is targeted to regulate *Eu. dicksonii* growth, temporarily silencing genes possibly as a stealth mechanism, upon onset of infection. Alternately, this could be the product of a generalized stress response in *Eu. dicksonii.*

Contig400862 and contig402793 also encode fragments of *Eu. dicksonii* translation factor(s) possibly involved in programmed cell death (Table S1). Neither contig is full-length and both are missing the predicted start of the gene(s), so it is not possible to predict the presence of signal peptides for secretion. The similar *P. infestans* protein (XP_002899252; PITG_14133) does not contain a signal peptide. Therefore Contig_400862 and contig_402793 may also represent oomycete gene(s) targeted internally rather than to the host.

#### Insights into pathogen physiology, stress responses and metabolism

We identified a unigene encoding a protein fragment predicted to be an hypoxia-induced protein. Contig_404084 from *Eu. dicksonii* was similar to HIGi hypoxia-inducible domain family member 2A from *Xenopus laevis* and the predicted protein fragment contained two transmembrane domains and an HIG_1_N response to hypoxia domain (Table S1). We also identified three sequences with thioredoxin-like domains within our pathogen dataset (Table S1). Several gene fragments with functional domains classified as transporters were also identified in *Eu. dicksonii* (Table S1). None of these sequences were full length, or predicted to contain signal peptides, however they may play an important role in the uptake of nutrients from the host.

One pathogen sequence, Contig_500199, predicted to encode a secreted lipase was identified in the dissected library, indicating host lipids may be an important nutrient source for *Eu. dicksonii* (Table S1). We identified 13 sequences, which we were unable to determine as secreted (1.3% of those with functional domains; 0.4% of total dataset) of *Eu. dicksonii* origin, that are involved in lipid metabolism, and 29 (2.3% of those with a functional domain and 1% of total dataset) involved in amino acid biosynthesis and metabolism (Figure 4), highlighting the importance of these processes in oomycete metabolism as previously reported in higher oomycetes [60], [61].

#### Host responses to Eu. Dicksonii

Contig1359 encodes the full-length putative protein inhibitor Esi0079_0059 from *E. siliculosus.* Esi0079_0059 contains a proteinase inhibitor I13 domain (Table S1) and is predicted to exhibit serine-type endopeptidase inhibitor activity. This protein also contains a signal peptide for secretion from host cells. Esi0079_0059, is therefore, likely to be secreted as a defence response to protect host proteins from *Eu. dicksonii* secreted peptidases.

Contig303625 encodes the fragment of an *E. siliculosus* nuclear movement domain protein (Esi0000_0481). This protein contains a p23_NUDC_like domain (Table S1), required for movement of the nucleus of other eukaryote species. Interestingly, the expression of this unigene correlates with a close association between the host nucleus and the infecting *Eu. dicksonii* thallus. The latter is observed at the earliest detectable stage of infection, suggesting an early migration of the host nucleus towards the infection site (Figure 1).

Contig2091 encodes an *E. siliculosus* protein (Esi0035_0036) currently un-annotated in the genome database. This protein contains a GRIM_19 domain, which promotes cell death via apoptosis in animals.

#### Expression of an E. siliculosus viral sequence

Intriguingly, Contig_403835 encodes a fragment of Esi0052_0171 a gene that belongs to the lysogenic virus inserted into the *Ectocarpus* genome. However, a comprehensive transcriptomic analysis reported by [5] revealed that the inserted viral genome is transcriptionally silent throughout the life cycle of *Ectocarpus*, even following the application of several stress treatments (hyperosmotic, hypoosmotic and oxidative stress). Hence, this result suggests that although lysogeny has yet to be observed in the *E siliculosus* strain used in the present study, the expression of inserted viral genes may still be triggered by infection from another pathogen. Unfortunately, the gene encoded by Esi0052_0171 (EsV-1-191) does not contain any conserved domains or have similarity to any functionally characterised protein, giving little clue as to what its function could be.

## Discussion

In this study we describe an extensive characterisation of an EST collection of the brown alga *E. siliculosus* infected with the marine oomycete *Eurychasma dicksonii*. Given its wide host range and worldwide distribution, *Eu. dicksonii* is likely to be important to the ecology of coastal habitats where brown algae are predominant primary producers. Furthermore, *Eu. dicksonii* belongs to the most early branching clade within the oomycete lineage, and is therefore of considerable interest to decipher the origin and evolution of pathogenicty in this group. This study represents the first large scale sequencing study undertaken outside the best studied oomycete orders (namely Pythiales, Peronosporales and Saprolegniales). The latter, however, only represent a fraction of the lineage's diversity. Thus, and perhaps not so surprisingly, only 1314 (42% of a total 3074) pathogen unigenes shared sequence similarity with known oomycete genes. 1760 of the pathogen unigenes had no similarity to any known sequence, and no defining protein domains with which to predict function. In a similar study of the *Aphanomyces euteiches* transcriptome, Gaulin et al [36] found that approximately 70% of EST sequences showed similarity to previously described genes in the NCBI non-redundant protein database and around 80% showed similarity to *Phytophthora* proteins. Within the sequences we identified of *Eu. dicksonii* origin, only a maximum of 28% showed similarity to previously described proteins. Therefore, our *Eu dicksonii* gene dataset is largely unique and indicates how far away we are from identifying the full repertoire of oomycete genes, despite the availability of several genomes from plant and fish pathogenic species. Additionally, it is clear from this study that *Eu. dicksonii* shares some genetic similarity with higher order oomycetes, but is in fact very unique genetically, in comparison to these other sequenced oomycetes.

Making use of the recently completed *E. siliculosus* genome [5], we were able to unambiguously map 6787 of our unigenes to the algal host. Of these sequences, only approximately 20% mapped to protein coding regions. *E. siliculosus* contains large 3′UTR regions and mapping of EST datasets generated in previous projects has resulted in large proportions mapping to these 3′ UTRS [5]. Therefore the result that up to 80% of our sequences map outside of the protein coding regions of *E. siliculosus* genes is consistent with previously reported data and may go part-way to explaining the low number of host sequences with significant similarity in the NCBI nr protein database.

The unusually high level of expression of a broad range of TEs in *E. siliculosus* is also fully consistent the observations reported by the *Ectocarpus* Genome Initiative [5]. Importantly however, the most highly expressed TEs in our libraries only partially overlap those identified in this earlier work. Our results suggest an environmental, most probably stress-dependent, transcriptional regulation of TEs in *E*.*siliculosus* We conclude that, whilst methylation has not been detected in *E. siliculosus*, TE expression is probably tightly regulated in the genome by an as yet uncharacterised mechanism. Finally, the fact that the retroelements RTE2, RTE3 and RTE4 are the most highly induced in infected libraries and among the most abundant repeats in the *E. siliculosus* genome points to a stress-induced transposing activity, which remains to be investigated.

We found a GC content in host sequences of 51.9% consistent with the *E. siliculosus* genome GC content of 53.6%. However, analysis of our *Eu. dicksonii* dataset revealed a much lower GC content, at 40.5%. This figure appears to be representative of real protein encoding *Eu. dicksonii* genes sequenced so far, and is not a result of poor quality or non-protein coding sequencing. This result is in stark contrast with the GC content of other sequenced oomycete genomes, which is approximately 58% GC [14], [28]–[30]. A breadth of possible explanations, including pathogenic lifestyle, have been formulated to account for low genome GC content [62]. Hence, the biological significance of the GC-impoverished *Eu. dicksonii* transcriptome remains unclear . Interestingly, it is known that unlike other oomycetes, *Eu. dicksonii* uses a previously reported stop codon to encode tryptophan in mitochondrial genes [7]. This bias towards the use of UGA, rather than UGG in mitochondria is consistent with the widely held view that AT-rich genomes are prone to Trp-UGA codon reassignment.

Several hundred candidate effector molecules carrying a RXLR or CRN motif have now been identified in the genomes of oomycetes pathogenic on plants [16], [28] and one putative RXLR-effector was found in *Saprolegnia parasitica*, which is a pathogen of fish [63]. It is widely assumed that the RXLR motif mediates the translocation of pathogenicity effectors into host cells [55], [64]. It is also functionally interchangeable with the PEXEL translocation signal of apicomplexa pathogens [65], [66]. Despite *Eu. dicksonii* being an obligate intracellular biotrophic pathogen, we were unable to unambiguously identify oomycete effector molecules in our dataset. Thus, it is possible that *Eu. dicksonii* does not contain RXLR proteins, and uses an alternate signal for translocation of effectors into host cells. Alternately, since the current study does not represent a complete *Eu. dicksonii* transcriptome, and since many of our sequences are not full length, RXLR effectors may be present, but unidentifiable using the current dataset. Furthermore, if such effector sequences are expressed at low levels we may not pick them up in EST libraries derived from infected host material. Full transcriptome or genome sequencing of *Eu. dicksonii* is therefore required to comprehensively search for the ancient origins of RXLR, CRN or other effectors within the oomycete lineage.

Whilst RXLR or CRN-type effectors were not identified in this study, we were able to identify sequences as potential pathogenicity factors, including both genes not previously described as oomycete pathogenicity determinants, and genes with similarity to pathogenicity factors from oomycetes and other organisms.

A novel putative pathogenicity factor is the protein encoded by Contig500758, a predicted alginate lyase. This sequence does not have homologues in sequenced oomycete genomes and appears to be most similar to alginate lyases from fungi and brown algal grazers such as abalone (*Haliotis* ssp., Figure 6). Since alginates are the major structural component of the brown algal cell wall, we hypothesize that this *Eu. dicksonii* protein may be a major pathogenicity determinant. Several other sequences with similarity to cell wall degrading enzymes were also identified in the pathogen dataset. It has been reported that *Eu. dicksonii* has an extremely wide host range, infecting all brown algae tested so far [6]. It is possible that this infection is achieved primarily through the pathogen's ability to degrade host alginate, and other cell wall structural components, and thereby enter the host cell. Several other proteins involved in production or degradation of both host and pathogen cell walls were also identified in this study, indicating the importance of the cell wall in oomycete-host interactions. Enzymes involved in alginate and cellulose biosynthesis were identified in the host sequence set, whilst a putative chitin synthase was identified in *Eu. dicksonii*. This suggests that chitin or chitosaccharides may be ancient and important components of the oomycete cell wall that may have either been lost in higher oomycetes, or which fulfil subtle functions in structuring the cell wall.

We identified three *E. siliculosus* putative cellulose synthases in our host dataset. Evidence for secreted cellulose degrading enzymes in *Eu. dicksonii* was not as conclusive. It is possible that *E. siliculosus* strengthens the cell wall using cellulose in response to pathogen attack and degradation of alginates. Esi0185_0053 has a CesA_CelA-like domain, and Esi0231_0017 has a cellulose synthase domain, along with a glycosyl transferase GTA family domain. However, Esi0004_0105 only contains a glycosyl transferase GTA family domain. Since the annotation of these genes is purely based on bioinformatic analysis, their exact enzymatic function is not known. Therefore, it is possible that at least one of these genes could function in the production of the related defence molecule callose, rather than cellulose.

Rearrangement of actin microfilaments to focus on the infection site, and movement of the host nucleus to the site of attack is a well-documented feature of plant-oomycete and plant-fungal interactions (reviewed in [67]). However, little is known of the brown algal response to infection. In the present study, we identified an *E. siliculosus* candidate nuclear movement protein within our dataset. Furthermore, the *Eu. dicksonii* thallus is always observed in close proximity to the host nucleus and Gogli, ([7]; Figure 1 this study). This association between *Eu. dicksonii* and its host nucleus is already established at the earliest detectable stages of infection, and is conserved across the brown algal genera *Ectocarpus*, *Macrocystis* and *Pylaiella*. These observations suggest that migration of the host nucleus towards the parasitic thallus is a physiologically-relevant feature underpinning the infection process.

Further host responses to infection by *Eu. dicksonii* might be mediated by the secretion of a proteinase inhibitor, presumably targeted towards proteinases produced by the pathogen. These findings highlight the power of the production of EST libraries from infected host material to build up a picture of the dynamics of the interactions between *E. siliculosus* and *Eu. dicksonii* during infection. However, since many of the oomycete sequences identified in this study do not show similarity to previously described genes, future challenges include functional characterisation of such genes and identification of further pathogenicity determinants in this organism.

## Supporting Information

Figure S1 ***Ectocarpus*** **assembled contigs.**
(TXT)

Figure S2 ***Eurychasma*** **assembled contigs.**
(TXT)

Table S1 **Sequences providing insights into** ***Eu. dicksonii*** **infection of** ***E. siliculosus.***
(XLS)
